# Achieving long cycle life for all-solid-state rechargeable Li-I_2_ battery by a confined dissolution strategy

**DOI:** 10.1038/s41467-021-27728-0

**Published:** 2022-01-10

**Authors:** Zhu Cheng, Hui Pan, Fan Li, Chun Duan, Hang Liu, Hanyun Zhong, Chuanchao Sheng, Guangjin Hou, Ping He, Haoshen Zhou

**Affiliations:** 1 Center of Energy Storage Materials & Technology, College of Engineering and Applied Sciences, Jiangsu Key Laboratory of Artificial Functional Materials, National Laboratory of Solid State Microstructures and Collaborative Innovation Center of Advanced Microstructures, Nanjing University, Nanjing, 210093 P. R. China; 2 Dalian National Lab for Clean Energy, State Key Laboratory of Catalysis, Dalian Institute of Chemical physics, Chinese Academy of Sciences, Dalian, 116023 P. R. China

**Keywords:** Batteries, Batteries

## Abstract

Rechargeable Li-I_2_ battery has attracted considerable attentions due to its high theoretical capacity, low cost and environment-friendliness. Dissolution of polyiodides are required to facilitate the electrochemical redox reaction of the I_2_ cathode, which would lead to a harmful shuttle effect. All-solid-state Li-I_2_ battery totally avoids the polyiodides shuttle in a liquid system. However, the insoluble discharge product at the conventional solid interface results in a sluggish electrochemical reaction and poor rechargeability. In this work, by adopting a well-designed hybrid electrolyte composed of a dispersion layer and a blocking layer, we successfully promote a new polyiodides chemistry and localize the polyiodides dissolution within a limited space near the cathode. Owing to this confined dissolution strategy, a rechargeable and highly reversible all-solid-state Li-I_2_ battery is demonstrated and shows a long-term life of over 9000 cycles at 1C with a capacity retention of 84.1%.

## Abstract (web summary)

Rechargeable Li-I_2_ battery system is interesting due to high theoretical capacities but the insoluble discharge product at the conventional solid interface leads to poor performances. Here, by using a confined dissolution strategy the authors demonstrate a rechargeable all-solid-state Li-I_2_ battery with extended cycle life.

## Introduction

State-of-the-art Li-ion battery based on intercalated cathode materials has been successfully applied in portable digital devices. However, the limited capacity and increasing cost of the intercalated cathode strongly restricts its further application in the expanding energy-storage market^1–3^. As an emerging novel battery system, the Li-I_2_ battery (LIB) has attracted great interest in recent years. Compared with conventional intercalated cathodes, the I_2_ cathode possesses many advantages, such as the high capacity of 211 mAh g^−1^, low cost, and environment-friendliness^4^. Most importantly, the high solubility of intermediate products (I_3_^−^), namely, polyiodides, in the liquid electrolyte can promote a fast electrochemical reaction for the I_2_ cathode^5^:

1 $$3{{{{{{\rm{I}}}}}}}_{2}+2{{{{{{\rm{e}}}}}}}^{-}\rightleftharpoons\, 2{{{{{{\rm{I}}}}}}}_{3}^{-}$$

2 $$2{{{{{{\rm{I}}}}}}}_{3}^{-}+4{{{{{{\rm{e}}}}}}}^{-}\rightleftharpoons\, 6{{{{{{\rm{I}}}}}}}^{-}$$

This phenomenon offers the liquid LIB a superior rate capability and long cycle life. However, during charging, the dissolved polyiodides would uncontrollably diffuse to the anode, causing a severe Li corrosion and a low coulombic efficiency. Such phenomenon is called the “shuttle effect”^6^. To address this tough issue, various carbon materials, such as hollow carbon fold-hemisphere^7^, heteroatom-doped carbon cloth^8^, and graphene layer^9^ have been developed to confine the iodide species within the porous structure. Nevertheless, the weak affinity between the polar polyiodides and porous carbon cannot totally prevent the dissolution of polyiodides. Therefore, the shuttle of polyiodides is inevitable in liquid LIB. In addition, safety concerns, such as leakage, fire, and explosion associated with liquid electrolytes, pose a huge challenge to the application of liquid LIBs^10^.

All-solid-state LIB based on nonflammable solid-state electrolyte can simultaneously solve the shuttle problem of polyiodides and the safety issue in liquid LIB. The primary all-solid-state LIB was commercialized to serve as the power source for pacemaker in 1972^11^, but it cannot be recharged and can only be discharged at low rates. In this primary LIB, as both the discharge product and solid-state electrolyte, LiI forms irreversibly upon discharge, leading to a large internal resistance and thus the failure of the battery. Li et al.^12^ tried to fabricate a rechargeable solid-state LIB by adopting LiI(3-hydroxypropionitrile)_2_ electrolyte. However, this battery can only be recharged for several cycles with limited capacity. To date, a reversible and long-life all-solid-state LIB is lacking because of following factors. First, contrary to a two-step polyiodide chemistry in liquid LIB, the I_2_ cathode undergoes a one-step I_2_/I^−^ redox reaction upon discharge in traditional solid-state LIB:

3 $${{{{{{\rm{I}}}}}}}_{2}+2{{{{{{\rm{e}}}}}}}^{-}\to 2{{{{{{\rm{I}}}}}}}^{-}$$

**Fig. 1:**
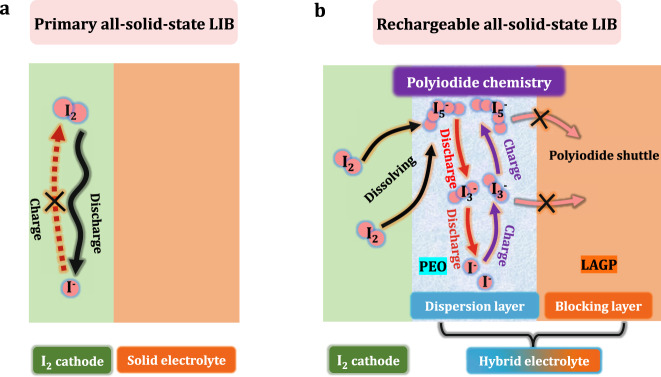
Schematic of the reaction mechanism at cathode/electrolyte interface in (a) the primary all-solid-state LIB and (b) a rechargeable all-solid-state LIB in this work. In the primary all-solid-state LIB, the solid-phase conversion process between I_2_ and I^−^ suffers from a sluggish kinetics and leads to a poor rechargeability for the battery. In our proposed rechargeable all-solid-state LIB, a two-step polyiodide chemistry with fast kinetics and high reversibility is realized by the confined dissolution strategy. The polyiodides could be well dissolved in the dispersion layer, but their shuttling is avoided by the blocking layer. During the discharge process the I_5_^−^ is first discharged to I_3_^−^ and then to I^−^. The charge process follows a conversion reaction from I^−^ to I_3_^−^ and finally I_5_^−^. Note that I_2_ accepts electron from KB to form I_5_^−^ and is then dissolved in the dispersion layer.

The solid-phase conversion leads to sluggish reaction kinetics and poor rechargeability for the battery as shown in Fig. 1a. Second, the intrinsically poor solid/solid interfacial contact and instability of the solid-state electrolyte cause considerable internal resistance for the battery. Therefore, to achieve a rechargeable and high-performance all-solid-state LIB, eliminating the traditional solid-phase reaction path is essential, and a new battery chemistry with fast kinetics and high reversibility should be designed.

Herein, a brand new and satisfactory battery chemistry is realized in the all-solid-state LIB by using a “confined dissolution” strategy. The core idea of this strategy is to localize the dissolution of polyiodides within the limited space near the I_2_ cathode. Meanwhile, the shuttle of polyiodides to the anode side is totally blocked. As illustrated in Fig. 1b, a hybrid electrolyte consisting of a dispersion layer and a blocking layer is elaborately designed. Poly(ethylene oxide) (PEO) acting as the dispersion layer could largely dissolve polyiodides generated during discharge/charge and thus promote a fast and highly reversible polyiodide chemistry. A single Li-ion conductor Li_1.5_Al_0.5_Ge_1.5_(PO_4_)_3_ (LAGP) as a blocking layer is adopted to effectively avoid the shuttle effect of polyiodides. In addition, the PEO dispersion layer has a low Young’s modulus. This could provide a close contact between the solid electrolyte and solid I_2_ cathode, facilitating a facile Li-ion transport within the cell. Thus, by employing this hybrid electrolyte structure, polyiodide redox reaction is successfully induced. Given the fast and stable polyiodide redox reaction, the all-solid-state LIB exhibits superior electrochemical performance and high safety, showing a huge application prospect for energy-storage requiring long cycle life and high safety.

## Results and discussion

### Characterization and electrochemical behavior of the I_2_ cathode

**Fig. 2:**
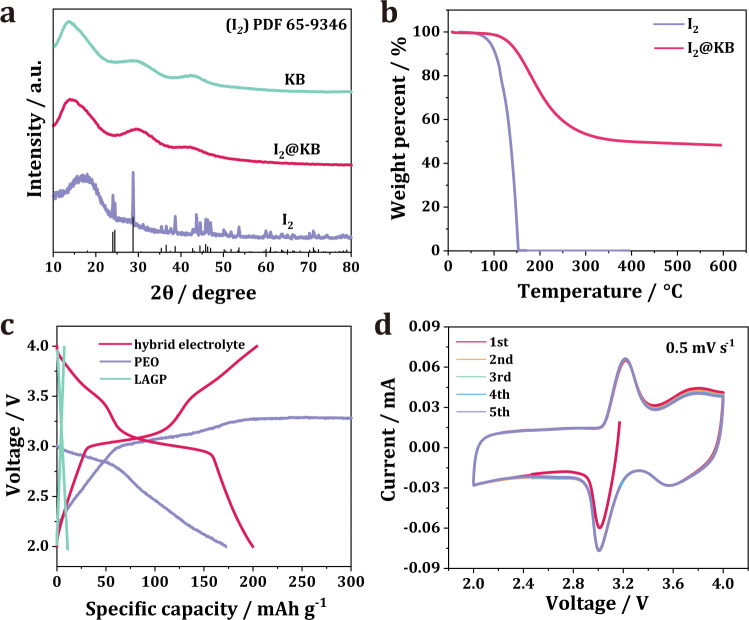
Characterization of I_2_@KB cathode and its electrochemical behavior. **a** XRD pattern of KB, I_2_ and I_2_@KB. **b** Thermogravimetric analysis curves of I_2_ and I_2_@KB. **c** The discharge/charge curves of all-solid-state LIB based on PEO electrolyte, LAGP electrolyte and hybrid electrolyte. **d** CV curves of all-solid-state LIB at sweeping rate of 0.5 mV s^−1^ for first 5 cycles.

Given that I_2_ is an electronic insulator and easy to sublimate, KB with high specific surface area and superior electronic conductivity was mixed with I_2_ by ball milling to prepare the I_2_@KB cathode. The scanning electron microscopy image and corresponding energy-dispersive spectra of the cathode demonstrated a uniform distribution of I_2_ within the KB framework (Supplementary Fig. 1). The X-ray diffraction (XRD) pattern in Fig. 2a shows that after ball milling, the peaks of I_2_ completely disappeared for the I_2_@KB cathode. This phenomenon was most likely caused by the successful impregnation of I_2_ into the KB porous structure. Thermogravimetric analysis was performed to determine the weight ratio of I_2_ in the I_2_@KB cathode, and the result is shown in Fig. 2b. Pure I_2_ started to lose weight before 75 °C and totally evaporated at 150 °C. After loading on KB, I_2_ could remain stable until 125 °C because of the strong absorption ability of KB. The I_2_ content in I_2_@KB was measured to be ~50%, which was higher than most reported I_2_ cathodes prepared by the solution–adsorption method^13–15^.

The I_2_ cathode suffers from a severe shuttle effect in liquid battery, because polyiodides could be easily dissolved in liquid electrolytes. Here, a hybrid electrolyte composed of PEO (dispersion layer) and LAGP (blocking layer) was employed to localize the polyiodide dissolution near the I_2_ cathode. To improve the contact between hybrid electrolyte and Li metal, a thin PEO layer was also introduced at LAGP/Li interface. As can be seen in Fig. 2c, the all-solid-state LIB based on the hybrid electrolyte exhibited an ideal columbic efficiency of 100%, with a capacity of ~200 mAh g^−1^ close to the theoretical value of the I_2_ cathode (211 mAh g^−1^). Note that the capacity contribution of KB and carbon cloth current collector were measured to be 10 mAh g^−1^ and 3 mAh g^−1^ (Supplementary Fig. 2), respectively. To verify the essential role of the hybrid electrolyte, all-solid-state LIBs without dispersion layer (only LAGP as solid electrolyte) and without blocking layer (only PEO as solid electrolyte) were assembled. Without the dispersion layer, the all-solid-state LIB could only deliver a capacity of 10 mAh g^−1^. This could be attributed to the sluggish I_2_/I^−^ solid conversion and a poor interfacial contact between LAGP and solid electrodes. By contrast, the battery without a blocking layer showed an endless charging plateau at 3.25 V, resembling that in liquid LIB. This phenomenon indicates that the polyiodides generating during the charge process uncontrollably shuttled back and forth between the cathode and anode due to the lack of blocking layer, and thus led to a low columbic efficiency. The above results strongly prove that the confined dissolution of polyiodides plays an important role in achieving high capacity and ideal columbic efficiency for the all-solid-state LIB.

It was interesting to find out that the discharge curve of the all-solid-state LIB showed two distinct plateaus at 3 V and 3.6 V, respectively, instead of a single slope reported in the previous solid-state LIB^12^. This indicates a new battery chemistry for all-solid-state LIB promoted by the hybrid electrolyte. The cyclic voltammetry (CV) results also confirmed a two-step reaction process (Fig. 2d). At a scan rate of 0.5 mV s^−1^, two pairs of redox couples located at 3.6/3.65 V and 3/3.2 V were observed, respectively. The pair of peaks at 3/3.2 V could be attributed to the I_3_^−^/I^−^ redox couple commonly reported in liquid LIBs^5^. Previous studies have limited the cut-off voltage below 3.6 V, and the other pair of redox couple at higher voltage (3.6/3.65 V) was usually incompletely displayed^8,16–18^. This redox couple was most likely corresponding to the I_5_^−^/I_3_^−^, which would be discussed in the following section of the reaction mechanism. From the second to the fifth cycle, the CV curves perfectly overlapped with each other, implying the highly reversible and stable electrochemical process of the battery.

### Reaction mechanism of the all-solid-state LIB

**Fig. 3:**
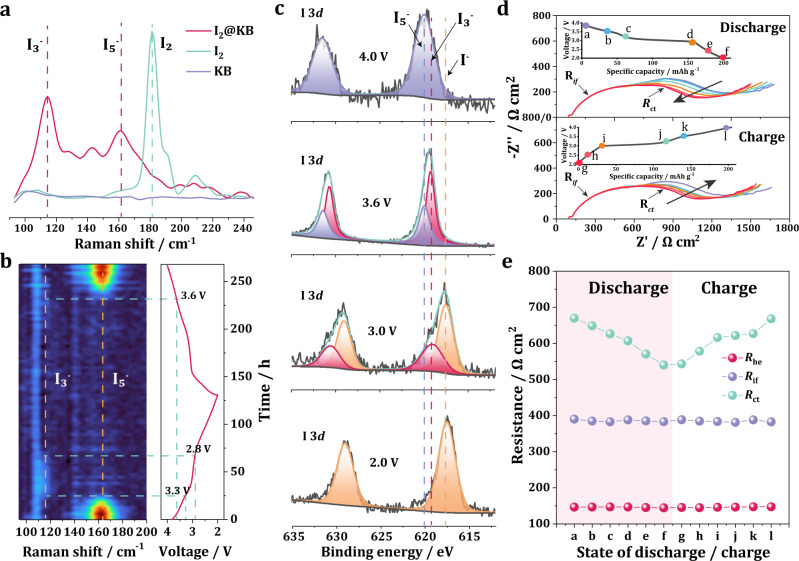
Reaction mechanism study of all-solid-state LIB. **a** Ex situ Raman spectra of pure I_2_, KB and I_2_@KB cathode. **b** In situ Raman spectra of I_2_@KB cathode at different discharge and charge states for the second cycle. **c** I 3*d* XPS spectra of the I_2_@KB cathode at different state of discharge. **d** EIS curves of the battery during different charge and discharge state. **e** The evolution of electrolyte resistance *R*_he_, interfacial resistance *R*_if_ and charge transfer resistance *R*_ct_ at different SOD/SOC shown in (**d**).

As the all-solid-state LIB showed a different battery chemistry from traditional solid-state LIB, a series of spectroscopy studies were conducted to understand the detailed reaction mechanism. Supplementary Fig. 3 shows the ex situ Raman spectra of pure I_2_, KB, and pristine I_2_@KB cathode. Obvious D band (~1360 cm^−1^) and G band (~1560 cm^−1^) signals could be found for KB and I_2_@KB, while a signal shift corresponding to I_2_ was observed for I_2_@KB compared with pure I_2_. A detailed view of the Raman spectrum in the range of 90−250 cm^−1^ is displayed in Fig. 3a. A sharp peak appearing at ~182 cm^−1^ was assigned to pure I_2_. However, in the spectrum of I_2_@KB, the signal intensity at 182 cm^−1^ decreased substantially, and two broad peaks at ~116 cm^−1^ and ~162 cm^−1^ appeared. Brus et al.^19^ reported that absorbed I_2_ could accept electrons from the carbon materials to form iodine anions and then react with excess neutral I_2_ to produce polyiodides (I_5_^−^ or I_3_^−^) with strong Raman signals at ~165 cm^−1^ (I_5_^−^) and ~108 cm^−1^ (I_3_^−^). Therefore, the peaks located at 116 cm^−1^ and 162 cm^−1^ were attributed to I_3_^−^ and I_5_^−^, respectively, which are derived from the interaction between I_2_ and KB in the as-prepared I_2_@KB cathode. This explains why the charging capacity (from I^−^ to I_5_^−^) was always higher than the discharge capacity (from I_5_^−^/I_3_^−^ to I^−^) for the first cycle (Supplementary Fig. 4). This phenomenon was usually ignored in the previous reported LIBs^5,20,21^.

To reveal the real-time transformation of the I_2_@KB cathode during the electrochemical process, in situ Raman measurement was performed on a homemade Li-I_2_ pouch cell (Supplementary Fig. 5) for the first two cycles. For the first cycle, I_3_^−^ (116 cm^−1^) clearly disappeared during discharge and I_5_^−^ (162 cm^−1^) appeared at the end of the charge process (Supplementary Fig. 6). Given that the first cycle is not reversible, a complete transformation between the iodine species is displayed by the Raman spectrum of the second cycle (Fig. 3b). During discharge, the signal of I_5_^−^ faded into the background at ~3.3 V, at which I_3_^−^ started to appear. The I_3_^−^ signal disappeared at 2.8 V, which was attributed to the transformation from I_3_^−^ to I^−^. For the following charge process, the I_3_^−^ signal was difficult to distinguish from the background noise, but the formation of I_5_^−^ could be clearly observed at 3.6 V. The signal located at ~110 cm^−1^ existed during the whole discharge/charge process, which was supposed to appear from the background. From the in situ Raman result, a two-step reaction mechanism during the electrochemical process for the rechargeable all-solid-state LIB can be proposed as follows:

4 $$3{{{{{{\rm{I}}}}}}}_{5}^{-}+2{{{{{{\rm{e}}}}}}}^{-}\,\rightleftharpoons\, 5{{{{{{\rm{I}}}}}}}_{3}^{-}$$

5 $$5{{{{{{\rm{I}}}}}}}_{3}^{-}+10{{{{{{\rm{e}}}}}}}^{-}\,\rightleftharpoons\, 15{{{{{{\rm{I}}}}}}}^{-}$$

In previous studies in which the LIBs were often operated in the voltage range of 2–3.6 V, only the I_3_^−^ signal was observed in their in situ Raman results^5,7^, indicating a capacity waste of reaction (4). Therefore, extending the cut-off voltage to 4 V was essential to fully release the capacity of the I_5_^−^/I_3_^−^ redox couple. In general, the ideal final charge product of the Li-I_2_ battery should be I_2_. The battery was further charged to 4.1 V which is much higher than the I_2_/I_3_^−^ redox potential (~3.6 V) to see whether the I_5_^−^ will be charged to I_2_. As shown in Supplementary Fig. 7, only a sharp peak located at 162 cm^−1^ corresponding to I_5_^−^ appears in the ex-situ Raman spectrum of charged I_2_@KB cathode. It can be infered that the strong electron withdrawing ability of I_2_ would continuously accept electrons from the KB host, leading to a final charge product of I_5_^−^.

Ex situ X-ray photoelectron spectroscopy (XPS) was adopted to provide a complementary information of the chemical changes on the I_2_@KB cathode (Fig. 3c). At a high oxidation state of 4 V, the strong peak corresponding to high-valence state iodine (I_5_^−^) appeared at ~620 eV in the I 3*d* spectrum. After discharging to 3.6 V at which a I_5_^−^/I_3_^−^ conversion was expected, two peaks located at 620 eV (I_5_^−^) and 619.1 eV (I_3_^−^) were observed, respectively. This confirmed the reduction of I_5_^−^ to I_3_^−^ at the higher discharge plateau around 3.6 V. The peak of I_5_^−^ vanished when the I_2_@KB cathode was discharged to the second plateau of ~3 V, while signals located at 619.1 eV (I_3_^−^) and 617.5 eV (I^−^) showed up, indicating a I_3_^−^/I^−^ conversion at this voltage. Further discharging the cathode to 2 V led to a completely I_3_^−^ reduction to I^−^. The above XPS result shows high consistency with the proposed reaction mechanism.

To investigate the evolution of charge transfer kinetics during the electrochemical process, electrochemical impedance spectroscopy (EIS) test was carried out at different state of discharge/charge (SOD/SOC), the results of which are shown in Fig. 3d. According to previous studies on all-solid-state lithium batteries^22,23^, semicircle in the high-frequency region (>10^5^ Hz) represented the bulk resistance of the hybrid electrolyte (*R*_he_). The semicircle in the frequency region of 501~10^5^ Hz was ascribed to interfacial resistance between solid electrolyte and solid electrodes (*R*_if_), while the semicircle in the frequency region of 10~316 Hz was corresponding to the charge transfer resistance (*R*_ct_). A prominent variation of the *R*_ct_ could be observed during the whole discharge/charge process, while *R*_he_ and *R*_if_ almost remained unchanged. At the initial SOD process (stage a in Fig. 3d, also see Supplementary Fig. 8a), a significant higher value of the *R*_ct_ (671.8 Ω cm^2^) than the *R*_ct_ (542.3 Ω cm^2^) for the end state of charge (stage f in Fig. 3d, also see Supplementary Fig. 8b) was obtained. This was most likely attributed to a larger charge transfer barrier of reaction (4) than that of reaction (5), and related to the higher ionic conductivity and smaller size of I^−^ than I_5_^−^^15,24^. The result of the detailed fitting of the EIS curves at different SODs/SOCs is listed in Supplementary Table 1. The evolution of the electrolyte resistance *R*_he_, interfacial resistance *R*_if_ and charge transfer resistance *R*_ct_ during discharge/charge is plotted in Fig. 3e. *R*_he_ and *R*_if_ almost remained unchanged during the whole discharge/charge process, implying a very stable structure of the hybrid electrolyte. A monotonically decreasing charge transfer resistance was found along the discharge process, corresponding to the transformation process from I_5_^−^ to I_3_^−^ then to I^−^. As expected, *R*_ct_ grew again in the following charge process. The above EIS result strongly proved a stable and reversible electrochemical process for the all-solid-state LIB.

### Electrochemical performance

**Fig. 4:**
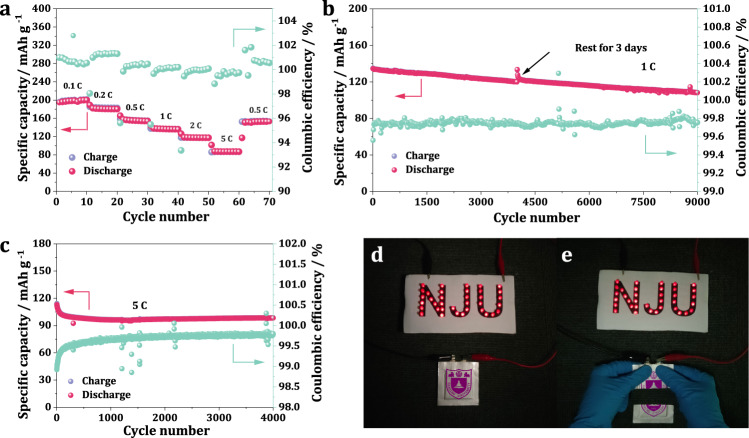
Electrochemical performance of all-solid-state LIB. **a** Rate performance of the as-designed all-solid-state LIB. **b** Cycling stability with the corresponding coulombic efficiency of the pouch type battery at 1C at a I_2_ mass loading of 0.5 mg cm^−2^. **c** Cycling stability with the corresponding coulombic efficiency of the all-solid-state LIB at 5C and at 90 °C. **d** Photograph of the all-solid-state Li-I_2_ pouch cell (40 mm × 40 mm cathode in 60 mm × 60 mm package) powering LED lights. **e** Illustration of the pouch cell showing high safety and well-running under a condition of being half-cut.

Although the LAGP blocking layer possesses a high ionic conductivity at room temperature, the high interfacial resistance between it and I_2_@KB cathode (Supplementary Fig. 9a) would severely limit the Li-ion transport, and thus lead to nearly no capacity for the battery based on LAGP electrolyte (Supplementary Fig. 9b). PEO with elastic property serving as both dispersion layer and buffer layer could greatly improve the interfacial contact between LAGP and I_2_@KB cathode (Supplementary Fig. 9a). As a result, the battery based on hybrid electrolyte showed a capacity of ~85 mAh g^−1^ at 30 °C (Supplementary Fig. 9c). This unsatisfying capacity was due to the relatively low room-temperature ionic conductivity of PEO. The specific capacity of the all-solid-state LIB was also measured at higher temperatures. As shown in Supplementary Fig 9c, the battery delivered specific capacities of 122, 175, and 202 mAh g^−1^ at 40, 50, and 60 °C, respectively. At a temperature of 60 °C, the battery resistance decreased significantly (Supplementary Fig 9d). Therefore, the following electrochemical performance was tested at 60 °C to obtain a better ionic transfer kinetics. During the rate performance test, the battery shows a specific capacities of 180.1, 159.9, 138.8, 117.5, and 83.2 mAh g^−1^ at 0.2, 0.5, 1, 2, and 5C, respectively (Fig. 4a). When the current was turned back to 0.5C, the specific capacity could recover to 158.8 mAh g^−1^. Given that the all-solid-state LIB showed excellent rate capability and high reversibility, discharge/charge cycling test at a high rate of 1C was conducted to verify the ultralong cycle life. The battery stably cycled for 9000 cycles, which lasted over 1 year, and maintained a high specific capacity of 112.9 mAh g^−1^, with a high capacity retention of 84.1% and an average coulombic efficiency up to 99.8% (Fig. 4b). The battery could still show high performance even after resting for 3 days. Such excellent long-term cycle stability should be attributed to the successful confined dissolution of polyiodides, which was proved by the XPS results shown in Supplementary Fig. 10. After long-term cycling the dissolution layer at the cathode side turned yellow, and clear polyiodide signals could be observed in the I 3*d* spectrum (Supplementary Fig. 10a). However, at the anode side, the protecting layer retained its original transparence (Supplementary Fig. 10b). In addition, no signal appeared in the I 3*d* spectrum, indicating that no polyiodide moved to the anode side. As a comparison, a LIB based on liquid electrolyte was assembled and showed endless charging behavior at the first charge process (Supplementary Fig. 11a), indicating a severe side reaction between shuttling polyiodides and Li metal. This could cause a serious Li anode corrosion and a capacity fading during cycling, as shown in the charge capacity cut-off cycling test for the liquid LIB (Supplementary Fig. 11b). A quick discharge capacity fading from 101 mAh g^−1^ to 56 mAh g^−1^ could be observed within only 100 cycles. The columbic efficiency also quickly dropped from 85.3% to 47.7%. These results provided strong evidence that the confined dissolution of polyiodides realized in the all-solid-state LIB is essential to achieve superior cycling stability.

In order to investigate the stability of the battery over long cycles, an EIS measurement was carried out for the all-solid-state LIB at the pristine state and after different cycles (Supplementary Fig. 12). Due to a solid electrolyte interface (SEI) formation process, an increased impedance could be observed for the initial cycles. From 20th cycles to 500th cycles, the battery displayed a negligible change of both *R*_he_, *R*_if_ and *R*_ct_, indicating a superior stability of the Li-ion transport within the battery. Moreover, a high areal capacity type all-solid-state LIB was designed to evaluate its application prospect. The mass loading of the active material was set as 8 mg cm^−2^, higher than most reported all-solid-state lithium batteries (usually < 1 mg cm^−2^). At 0.1C the battery could deliver a high specific capacity of 187.8 mAh g^−1^, corresponding to an areal capacity of 1.5 mAh cm^−2^ (Supplementary Fig. 13a), and still kept a capacity of 90 mAh g^−1^ after 100 cycles at 0.5C (Supplementary Fig. 13b).

To demonstrate the application of the all-solid-state LIB at higher temperatures, a long-term cycling at 90 °C was conducted. The battery delivered a specific capacity of 117 mAh g^−1^ at 5C and a capacity retention of 83.5% after 4000 cycles (Fig. 4c). The capacity fading at initial cycles was most likely attributed to the SEI formation process, which led to an increase for the battery resistance (Supplementary Fig. 14). Moreover, a 40 mm × 40 mm Li-I_2_ pouch cell (Supplementary Fig. 15a) was assembled to testify its safety performance. This single layer pouch cell was composed of a 40 × 40 mm cathode (210.5 mg total mass, 52.6 mg I_2_ mass), a 40 mm × 40 mm hybrid electrolyte of 326 μm thick (Supplementary Fig. 16) and a 40 × 40 mm Li foil of 100 μm thick. The pouch cell possessed a high specific capacity of 190 mAh g^−1^, corresponding to a capacity of 10 mAh (Supplementary Fig. 15b). As displayed in Fig. 4d, this pouch cell could readily power LED lights. Even after being cut into two pieces and resting in an argon-filled glove box for one week, the pouch cell could still run stably and power the LED lights (Fig. 4e). The above electrochemical performance and safety tests demonstrate a great application prospect of the all-solid-state LIB.

### Reaction kinetics and ion transport

**Fig. 5:**
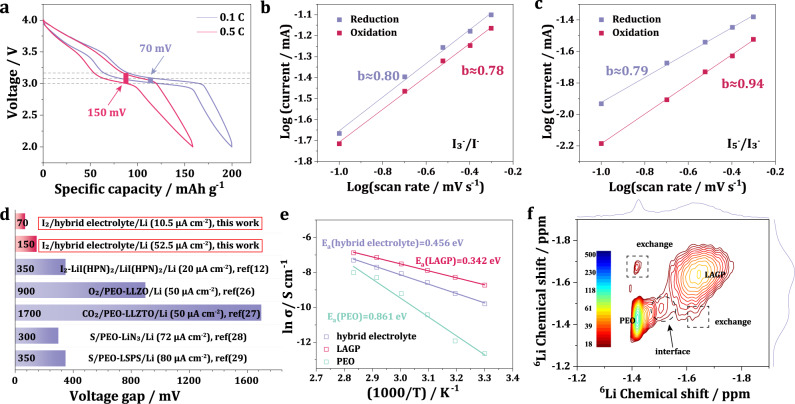
Reaction kinetics and Li ions transport property in the all-solid-state LIB. **a** Voltage gap of the all-solid-state LIB at 0.1C and 0.5C. **b**, **c** Plots of log(*i*) versus log (*v*) at specific peak currents extracted from the CV scans of (**b**) I_3_^−^/I^−^ and (**c**) I_5_^−^/I_3_^−^ redox couples. **d** Literature survey showing the voltage gap of all-solid-state batteries based on typical anion redox chemistry type cathodes, including Li-I_2_ battery based on LiI(HPN)_2_ electrolyte^12^, Li-O_2_ battery based on PEO-LLZO (Li_7_La_3_Zr_2_O_12_) composite electrolyte^26^, Li-CO_2_ battery based on PEO-LLZTO composite electrolyte^27^, Li-S battery based on PEO-LiN_3_ composite electrolyte^28^ and Li-S battery based on PEO-LSPS (Li_10_SnP_2_S_12_) composite electrolyte^29^. **e** Li-ion transport activation energy in PEO, LAGP and hybrid electrolyte calculated from EIS measurements. **f** 2D exchange ^6^Li NMR spectrum of the mixture of PEO and LAGP for a t_mix_ of 0.1 s.

In addition to the excellent electrochemical performance demonstrated above, it should be pointed out that the all-solid-state LIB showed a super low voltage gap during cycling. This contributed to a very high electrical energy efficiency for the battery. Compared to a big voltage gap of 350 mV at 20 μA cm^−2^ for a reported solid-state LIB^12^, the battery in this work showed a much lower voltage gap of 70 mV at 0.1C (10.5 μA cm^−2^) and of 150 mV at 0.5C (52.5 μA cm^−2^) (Fig. 5a). This should be attributed to the new polyiodides chemistry which has a much faster reaction kinetics than conventional solid-phase reaction chemistry. CV experiments at various scan rates were performed to investigate the reaction kinetics for the all-solid-state LIB (Supplementary Fig. 17). As the scan rate increased from 0.1 mV s^−1^ to 0.5 mV s^−1^, the gap between the reduction and oxidation potentials became larger due to the polarization, but two pairs of redox peaks were still well-defined. The relationship between the peak currents and scan rates could be determined as follows^25^: *i* = *av*^*b*^, where *i* and *v* stand for the peak current and corresponding scan rate, respectively; and *a* and *b* are adjustable parameters. The logarithm of this equation is log(*i*)* = b* log(*v*)* + *log(*a*), where *b* represents the domination degree of the ionic diffusion for the electrochemical process. If *b* is equal to 0.5, the ionic diffusion governs the electrochemical process. If *b* is equal to 1, a dominant capacitive-like behavior is indicated. As shown in Fig. 5b,c, good linear relationship between log(*i*) and log(*v*) was obtained for I_3_^−^/I^−^ and I_5_^−^/I_3_^−^ redox couples. The b-values were calculated to be 0.78 and 0.8 for the oxidation/reduction process of the I_3_^−^/I^−^ redox couple, and the corresponding values were 0.94 and 0.79 for the I_5_^−^/I_3_^−^ redox couples. Therefore, the redox reactions of I_3_^−^/I^−^ and I_5_^−^/I_3_^−^ were controlled by a combination of ionic diffusion and capacitive-like behavior. This capacitive contribution of the redox reaction is widely reported in liquid LIBs, which promoted a low voltage gap and a superior rate capability for the all-solid-state LIB. We further compared the voltage gap of all-solid-state LIB with three kinds of popular all-solid-state batteries based on conversion-type cathodes (S, O_2_, and CO_2_). It should be noted that the working areal current density for these batteries were comparable (20~80 μA cm^−2^). As shown in Fig. 5d, huge voltage gap of over 300 mV was observed for all-solid-state Li-S batteries regardless of the type of electrolyte used. For all-solid-state Li-O_2_ and Li-CO_2_ battery, the voltage gap could dramatically increase to 900 and 1700 mV, respectively. Such large voltage gap would lead to a low electrical energy efficiency, which becomes a big concern for their practical application.

Stable and facile Li-ion transport within the battery is another important reason for the low voltage gap. A Li–Li symmetric cell based on the hybrid electrolyte showed a low overpotential of ~45 mV and outstanding stability over 2000 h at a current density of 0.1 mA cm^−2^ (Supplementary Fig. 18). Besides the common cathode/solid electrolyte interface, the different natures between the dispersion layer and blocking layer led to the formation of a PEO/LAGP interface, which could have impact on the transport of Li ions. To gain a clear insight of the influence of the PEO/LAGP interface, EIS and solid-state nuclear magnetic resonance (NMR) measurements were performed for PEO, LAGP, and hybrid electrolyte. Activation energy of 0.342 eV was obtained from the EIS result for the Li-ion moving in LAGP (Fig. 5e). A similar value of 0.368 eV was calculated for LAGP from the NMR spin-lattice relaxation measurement (Supplementary Fig. 19), proving the reliability of the EIS results. At the presence of the PEO/LAGP interface, the activation energy of the hybrid electrolyte only slightly increased to 0.456 eV compared with that of LAGP, and this value was considerably lower than that for PEO (0.861 eV). This result indicates that the dissolution layer had minimal impact on the Li-ion transport. Then, 2D NMR exchange experiment was conducted to investigate the Li-ion exchange within the hybrid electrolyte. The 2D exchange NMR firstly records the resonance of ^6^Li atoms. After a certain mixing time (t_mix_) during which the ^6^Li atoms spontaneously exchange with other ^6^Li atoms in different chemical environments, the spectrum of the same ^6^Li atoms is recorded again. Therefore, in the 2D exchange spectrum, the off-diagonal signal represents the ^6^Li atoms, which have exchanged between two different environments. As shown in Fig. 5f, a significant Li-ion exchange in the off-diagonal area was observed for a t_mix_ of 0.1 s, proving a fast and spontaneous Li-ion transport between the PEO and LAGP. It should be noted that a resonance located at −1.47 ppm appeared in both 1D (Supplementary Fig. 20) and 2D NMR exchange spectra. This signal most likely arose from the Li chemical environment of the interface areal between PEO and LAGP. The interface area between inorganic particles and the polymer matrix has been reported to provide an additional fast Li-ion transport pathway^30^. In the hybrid electrolyte, facile Li-ion transport could also be achieved at the PEO/LAGP interface, which explains the reasonable activation energy of the hybrid electrolyte. Therefore, the rational design of the hybrid electrolyte played an important role in achieving fast polyiodides reaction kinetics and facile Li-ion transport within the all-solid-state LIB.

In summary, by using a confined dissolution strategy enabled by the well-designed hybrid electrolyte, a new polyiodides chemistry was successfully induced in the all-solid-state LIB. The dispersion layer promoted a fast and highly reversible redox reaction, while the blocking layer effectively localized the dissolution of polyiodides near the I_2_ cathode. The in situ Raman results revealed a two-step polyiodides reaction realized by I_5_^−^/I_3_^−^ and I_3_^−^/I^−^ redox couples during charge/discharge, instead of the conventional one-step solid-phase reaction (I_2_/I^−^). A fast reaction kinetics and facile Li-ion transport were also achieved as proved by the CV, NMR and EIS results. Therefore, the battery exhibited a high electrical energy efficiency, excellent rate performance, and a long cycle life of over 9000 cycles with 84.1% capacity retention at 1C. Moreover, the battery showed high safety and superior high-temperature performance. This work demonstrates a promising application potential of the all-solid-state LIB for energy-storage requiring high capacity and high safety, and opens a new avenue for the development of novel rechargeable all-solid-state batteries.

## Methods

### Material synthesis

Ball milling was used to prepare the I_2_@KB powder. I_2_ (Aladdin) and KB (EC-600JD) with a weight ratio of 1:1 was ball milled at 400 rpm for 4 h. To prepare the I_2_@KB cathode, PEO, LiTFSI serving as both the binder and ionic conduction component were mixed with I_2_@KB powder and then added into deionized water and stirred for 2 h to form a uniform slurry. The slurry was painted on a commercial carbon cloth or pressed on a stainless steel mesh (for high loading test) and then heated at 50 °C for 48 h to totally evaporate the water solvent. To obtain a better ionic transport within high loading cathode, 10 wt% succinonitrile solid plastizer was added into the cathode slurry. The I_2_ mass loading was ~0.5 mg cm^−2^ or 8 mg cm^−2^ (for the high mass loading test). The LAGP electrolyte was synthesized by pressing the LAGP powder (MTI) into a pallet with a diameter of 20 mm and then annealing it at 900 °C for 6 h. The hybrid electrolyte was prepared by dropping the PEO solution (PEO: bistrifluoromethanesulfonimide lithium salt (LiTFSI) = 18:1 in acetonitrile) on the surface of LAGP. Then, the hybrid electrolyte was heated at 60 °C in vacuum for 24 h. To improve the interfacial contact and stability between the LAGP and Li metal, a thin PEO layer was introduced at the LAGP/Li interface.

### Material characterizations

Thermogravimetric analysis was performed in DSC (TA SDTQ600, TA Instruments) from room temperature to 600 °C at a heating rate of 10 °C/min under nitrogen atmosphere. Raman tests were conducted on a NTEGRA Spectra AFM Raman Confocal SNOM instrument. A homemade cell with a quartz window was used for in situ Raman measurement. Electrochemical impedance spectroscopy (EIS) was carried out on an impedance analyzer (Solartron 1287 coupled with Solartron 1260). The perturbation voltage of 5 mV in the frequency range of 1 MHz to 0.1 Hz was applied. The EIS experiment data were interpreted using the ZPlot software. XRD test was investigated in PHI 5000 VersaProbe (Ulvac-Phi Co.). SEM characterization was conducted on a Hitachi SU8010 scanning electron microscope. XPS measurement was carried out on PHI5000 VersaProbe-II. Solid-state NMR measurement was carried out on a Bruker AVANCE III 400 MHz NMR spectrometer using a 4 mm DVT MAS NMR probe from room temperature to 65 °C, with ^6^Li and ^7^Li Larmor frequencies of 73.60 MHz and 155.5 MHz, respectively. The samples were packed into the center of the rotors, and the spinning rate was set to 8–10 kHz.

### Cell assembly and electrochemical tests

The all-solid-state LIB composed of Li metal anode, hybrid electrolyte (20 mm diameter), and I_2_@KB cathode. Then the battery were sealed by an aluminium-plastic film with nickel tab as anode current collector and aluminum tab as cathode current collector. As for the large size pouch cell, the I_2_@KB cathode (40 mm × 40 mm), a LAGP pellet (40 mm × 40 mm, Shenzhen MTI) with thin PEO layers, and Li metal (40 mm × 40 mm) were sealed in an aluminium-plastic package (60 mm × 60 mm). For the all-solid-state LIB based on PEO electrolyte, the PEO solution was cast on a Teflon plate and dried at 60 °C for 24 h to obtain a PEO film, which was directly used as the electrolyte. For the liquid LIB, the I_2_ cathode consisted of poly(tetrafluoroethylene) binder and I_2_@KB powder (mass ratio of 1:9), and the electrolyte was 1 M LiTFSI in 1,3-dioxolane/1,2-dimethoxyethane (DOL/DME) with 1 wt% LiNO_3_. All cell assembly processes were carried out in an argon-filled glove box. The discharge and charge tests of the all-solid-state LIB were carried out on a HJ1001SD8 (Hokuto Denko Corporation). The CV measurements were performed on an impedance analyzer (Solartron 1287 coupled with Solartron 1260).

## Supplementary information

Supplementary Information

## Data Availability

All data that support the plots within this paper and other findings of this study are available from the corresponding author upon reasonable request.
