# Supplementary material for: Achieving long cycle life for all-solid-state rechargeable Li-I2 battery by a confined dissolution strategy
Source: Nat Commun. 2022 Jan 10;13:125. doi: 10.1038/s41467-021-27728-0 (PMC8748797; doi:10.1038/s41467-021-27728-0)
Supplement: Supplementary file 1 — Supplementary Information [file 41467_2021_27728_MOESM1_ESM.pdf]

Supplementary Information

Achieving long cycle life for all-solid-state rechargeable Li-I_2 battery by a confined dissolution strategy

Zhu Cheng[1], Hui Pan[1], Fan Li[2], Chun Duan[1], Hang Liu[1], Hanyun Zhong[1],Chuanchao Sheng[1], Guangjin Hou[2], Ping He[1*] and Haoshen Zhou[1*]

[1]Center of Energy Storage Materials & Technology, College of Engineering and Applied Sciences, Jiangsu Key Laboratory of Artificial Functional Materials, National Laboratory of Solid State Microstructures and Collaborative Innovation Center of Advanced Microstructures, Nanjing University, Nanjing 210093 (P. R. China)

[2]Dalian National Lab for Clean Energy, State Key Laboratory of Catalysis, Dalian Institute of Chemical physics, Chinese Academy of Sciences, Dalian 116023 (P. R. China)

* E-mail: pinghe@nju.edu.cn; hszhou@nju.edu.cn

Supplementary Figures

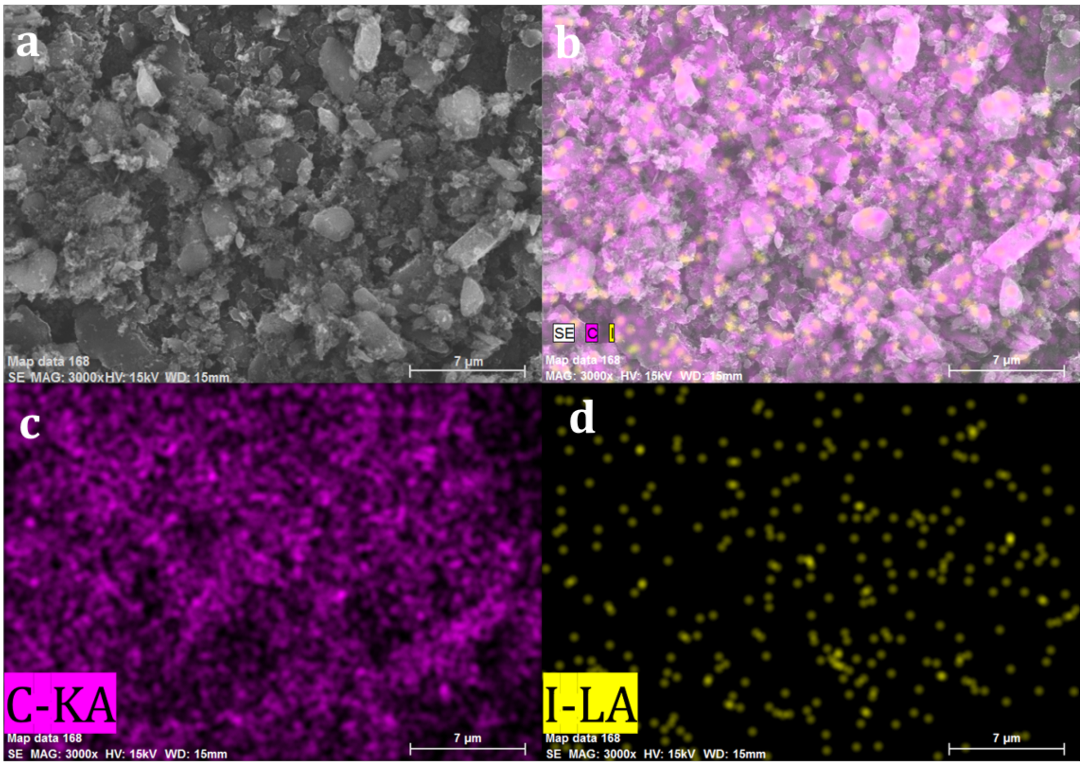

Supplementary Fig. 1 (a) SEM image of I_2@KB cathode. (b-d) EDS mapping of I_2@KB cathode, showing that I_2 was uniform distributed within the framework of KB.

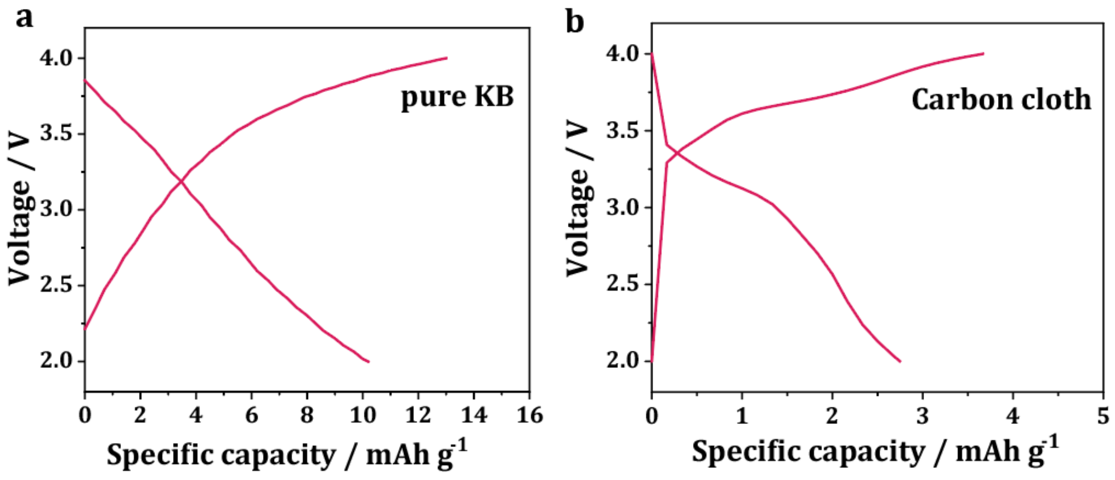

Supplementary Fig. 2 Voltage profiles of all-solid-state batteries based on (a) pure KB and (b) carbon cloth cathodes. The specific capacity of carbon cloth (12 mm diameter) is calculated based on the typical I_2 mass (0.56 mg) on the I_2@KB electrode of 12 mm diameter.

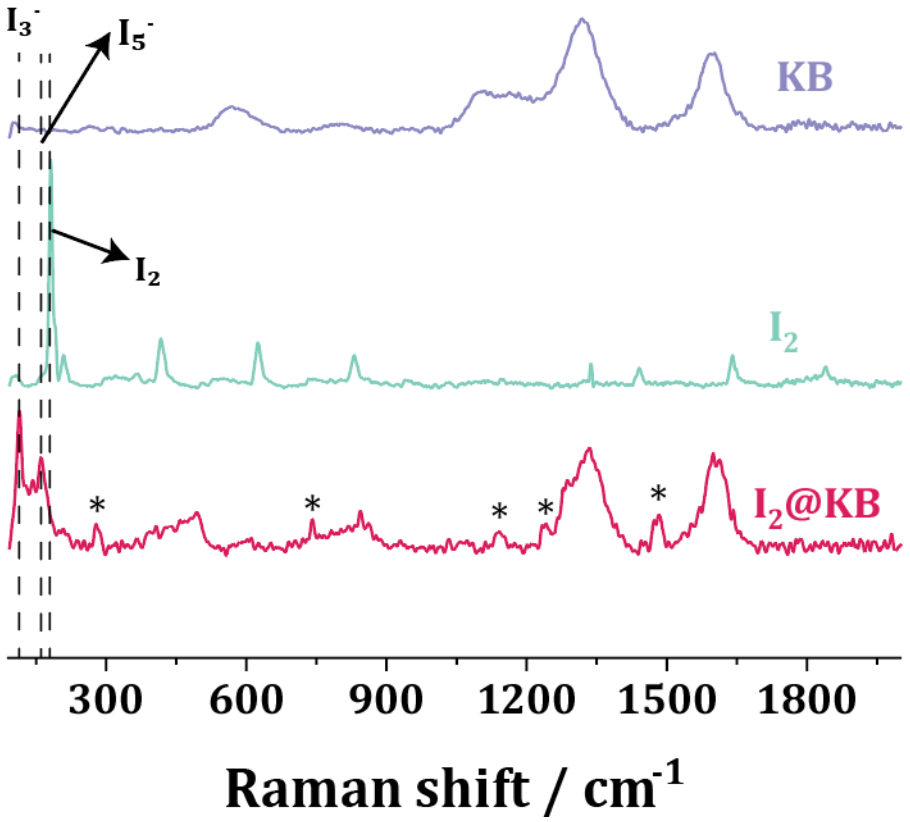

Supplementary Fig. 3 Raman spectra of KB, I_2 and pristine I_2@KB cathode in the range from 90 cm^{-1} to 2050 cm^{-1}. The Raman signals marked with asterisk correspond to PEO/LiTFSI within the cathode. Because I_2 could accept electrons from carbon materials to form iodine anions, and then reacts with excess neutral I_2 to produce I_5^- or I_3^-, the Raman signal of I_2@KB shift from 182 cm^{-1} (I_2) to 162 cm^{-1} (I_5^-) and 116 cm^{-1} (I_3^-).

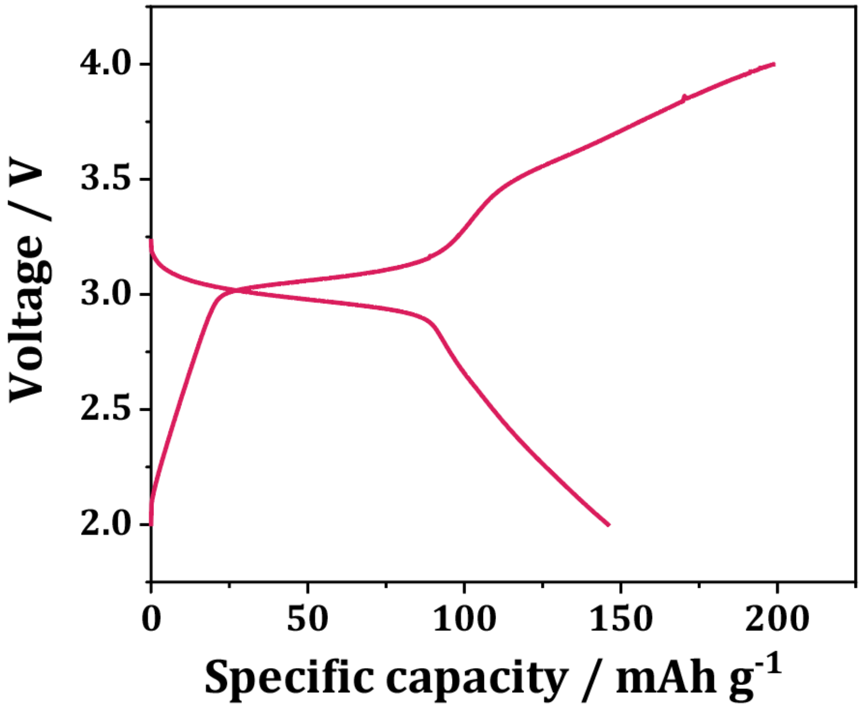

Supplementary Fig. 4 Typical voltage profile of the first cycle for all-solid-state LIB at 0.1 C. Because I_2 could accept electrons from KB to generate I_5^- and I_3^-, this could lead to a higher capacity for the charging process (from I^- to I_5^-) than the discharge process (from I_5^-/I_3^- to I^-) for the first cycle.

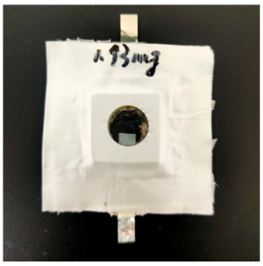

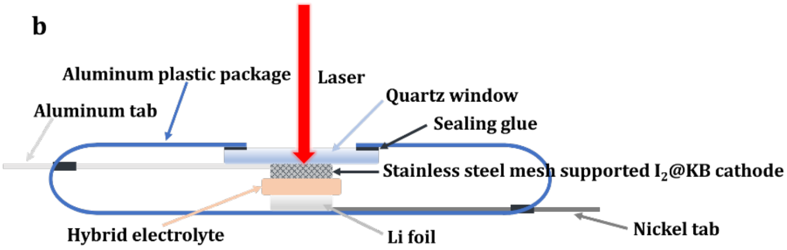

Supplementary Fig. 5 (a) The digital photo and (b) a diagram of the homemade all-solid-state Li-I_2 pouch cell for in-situ Raman measurement.

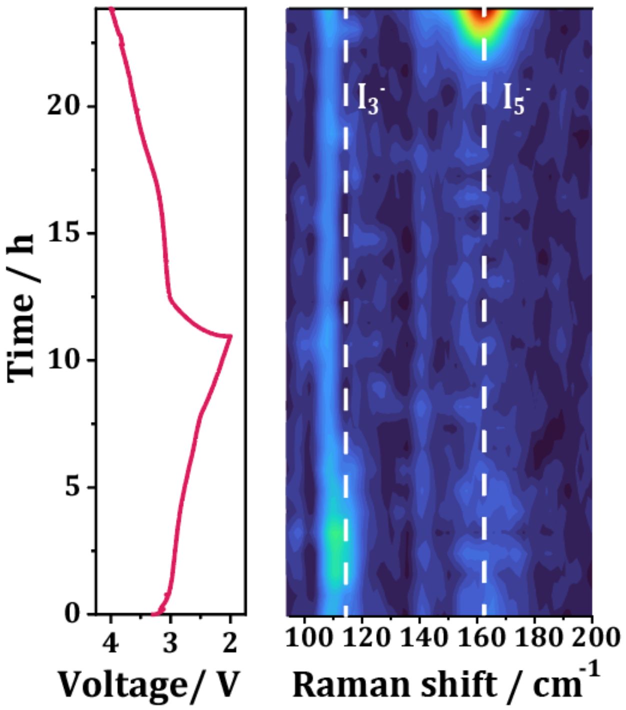

Supplementary Fig. 6 In-situ Raman result of the all-solid-state LIB for the first cycle, showing weak signals for I_3^- and I_5^- at the initial state and strong I_5^- signal after charging to 4 V. This suggests that the first cycle of the all-solid-state LIB is not a reversible process.

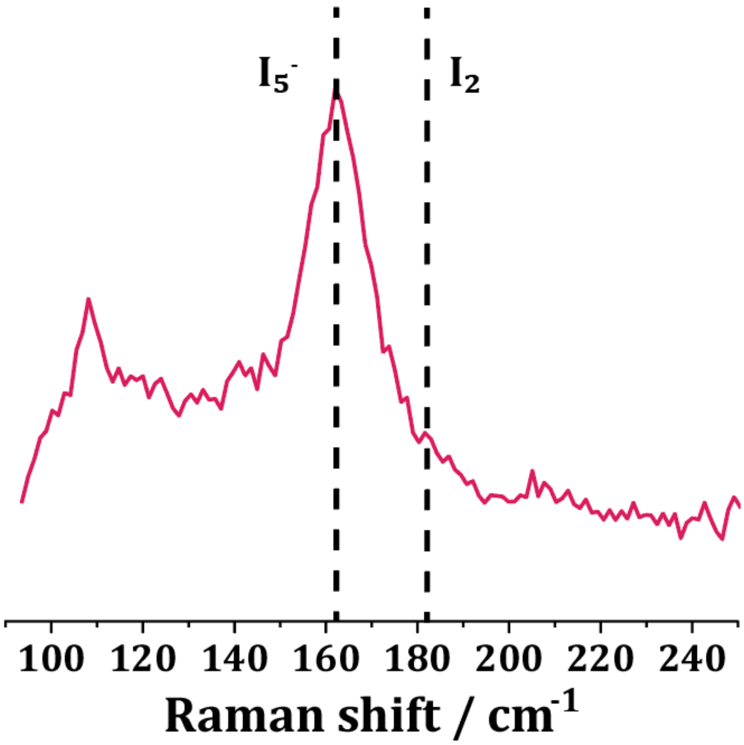

Supplementary Fig. 7 The ex-situ Raman spectrum of the I_2@KB cathode after charging to 4.1 V. Only a signal of I_5^- appeared in the spectrum, proving that the strong electron withdrawn ability of the iodine led a final charge product of I_5^-.

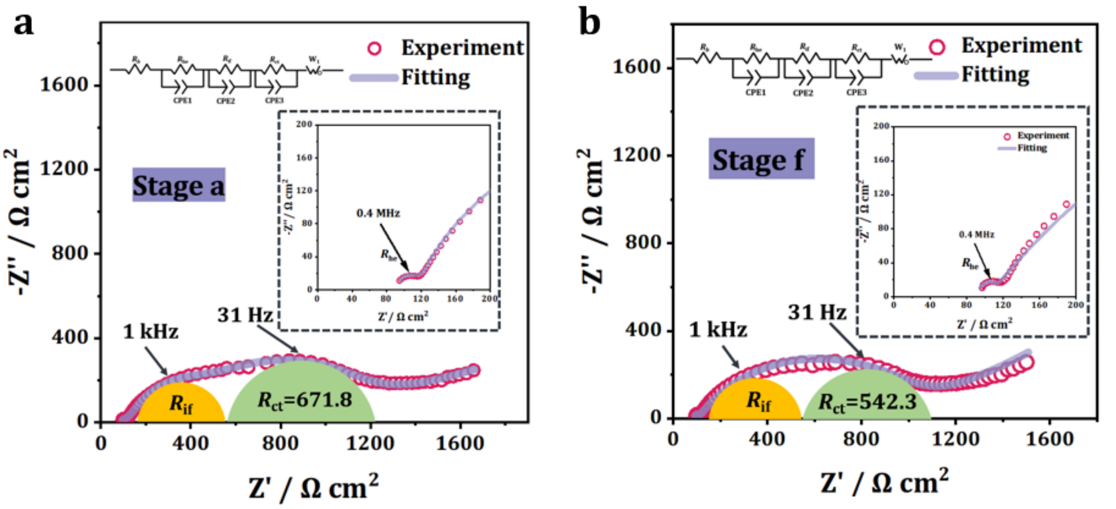

Supplementary Fig. 8 EIS result and the corresponding fitting of the all-solid-state LIB at a state of discharge corresponding to (a) stage a and (b) stage f shown in Fig. 3d. Insets are the zoomed EIS curves in high frequency range. The equivalent circuit for this EIS result is also shown in the figure. Two semicircles in middle frequency range were attributed to interfacial resistance (R_{if}) and charge transfer resistance (R_{ct}) respectively. The R_{ct} (671.8 Ω cm^2) for stage a (I_5^-) is significant higher than the R_{ct} (542.8 Ω cm^2) for stage f (I^-). The interface resistance was almost the same for these two stages, indicating a very stable electrolyte structure.

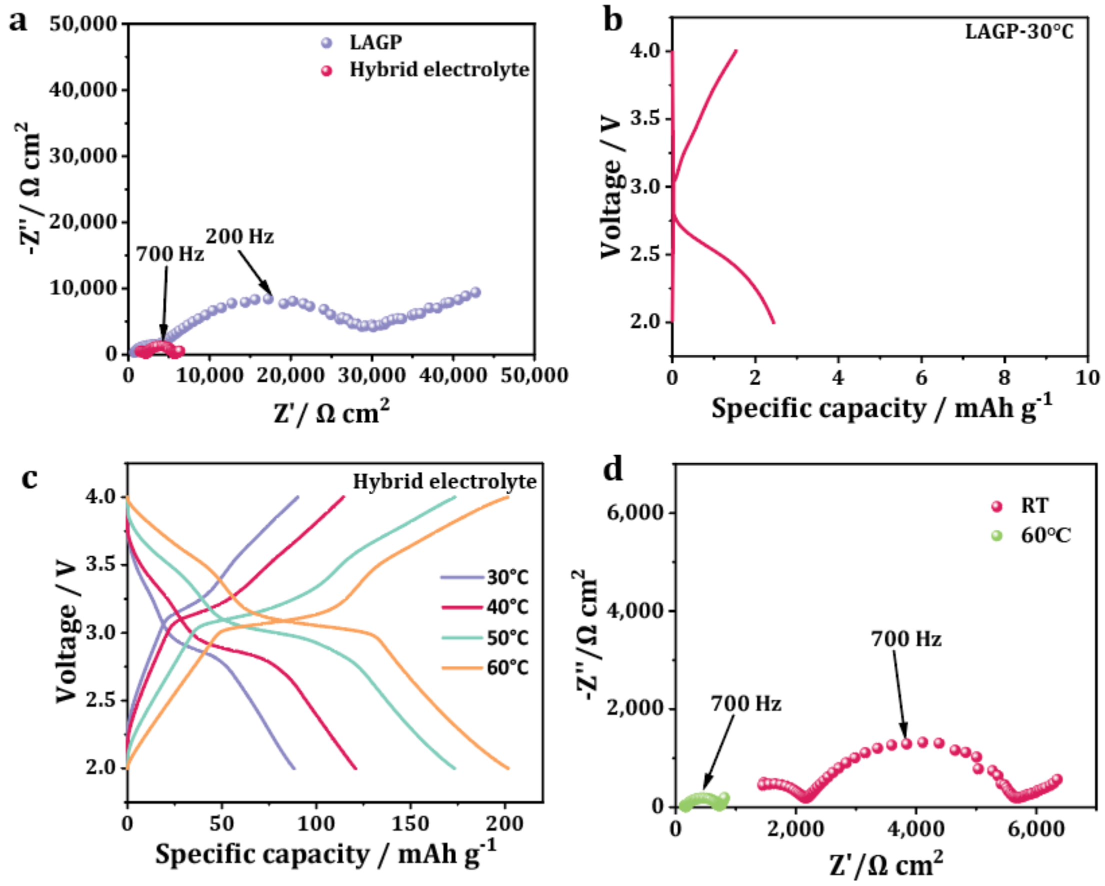

Supplementary Fig. 9 (a) EIS result of the all-solid-state LIB based on LAGP electrolyte and hybrid electrolyte at room temperature. The poor contact between cathode and LAGP is greatly improved by the introduction of PEO. (b) Voltage profile of the all-solid-state LIB based on LAGP at 30°C. (c) Voltage profile of the all-solid-state LIB at temperatures of 30, 40, 50 and 60°C. The battery showed specific capacities of 80, 122, 175 and 202 mAh g^{-1} at 30, 40, 50, and 60°C, respectively, proving an accelerated Li-ion transport at high temperatures. (d) EIS result of the all-solid-state LIB based on hybrid electrolyte at room temperature and 60°C. Increasing the working temperature to 60°C significantly improves the ion transfer in the battery.

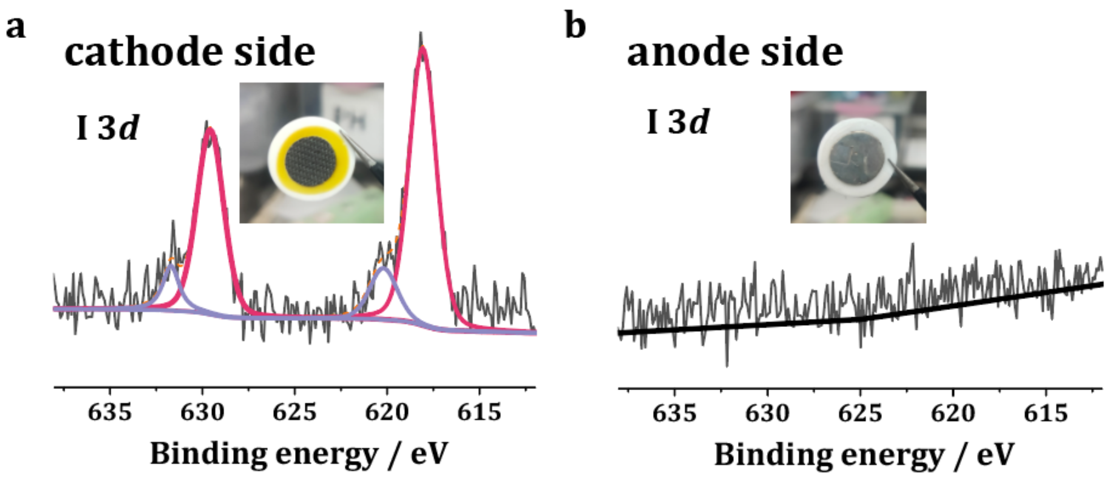

Supplementary Fig. 10 High-resolution I 3*d* XPS spectra of (a) dispersion layer at cathode side and (b) the PEO protecting layer at anode side in all-solid-state LIB after cycling. The insets in (a) and (b) show the different colors of PEO at (a) cathode and (b) anode side. Because LAGP only allows the transport of Li ions, polyiodides dissolved in cathode side could not shuttle to anode side. Therefore, no signal corresponding to iodine species was observed at Li metal side in the XPS spectrum.

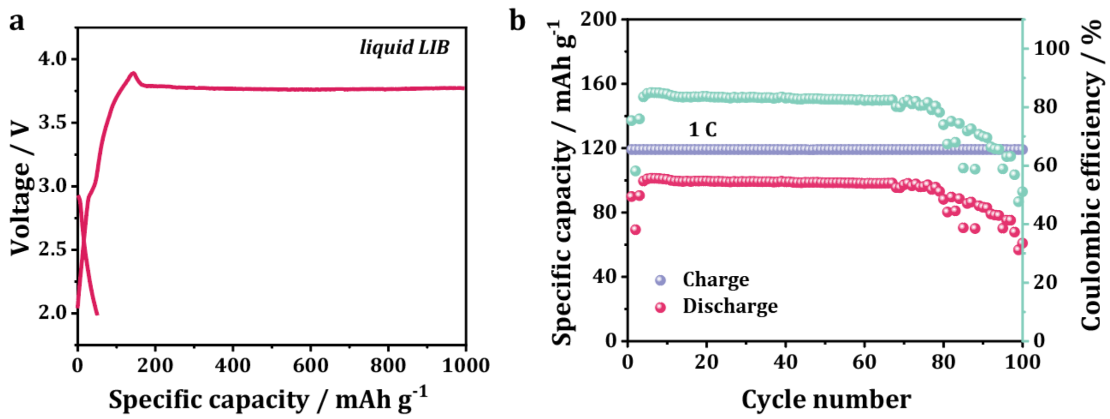

Supplementary Fig. 11 (a) First cycle voltage profile of liquid LIB at 1 C. (b) Cycling stability with the corresponding coulombic efficiency of the liquid LIB at 1 C. The charge capacity was controlled as 122 mAh g^{-1}. The battery showed endless charging behavior for the first cycle, indicating the severe shuttle effect of polyiodides. A severe capacity fading and a quick columbic efficiency drop were observed for the liquid LIB within just 100 cycles.

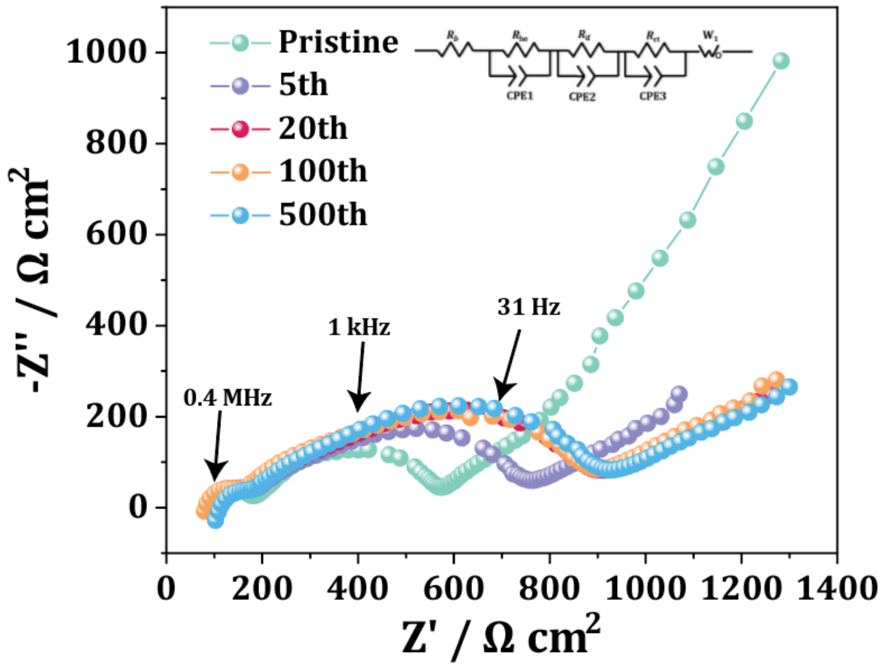

Supplementary Fig. 12 EIS plots of the all-solid-state LIB at pristine state and after 5, 20, 100 and 500 cycles. The battery impedance increased for the initial cycles due to a SEI formation process. After 20 cycles the overall impedance almost remains unchanged, indicating a very stable battery structure and highly reversible reaction process.

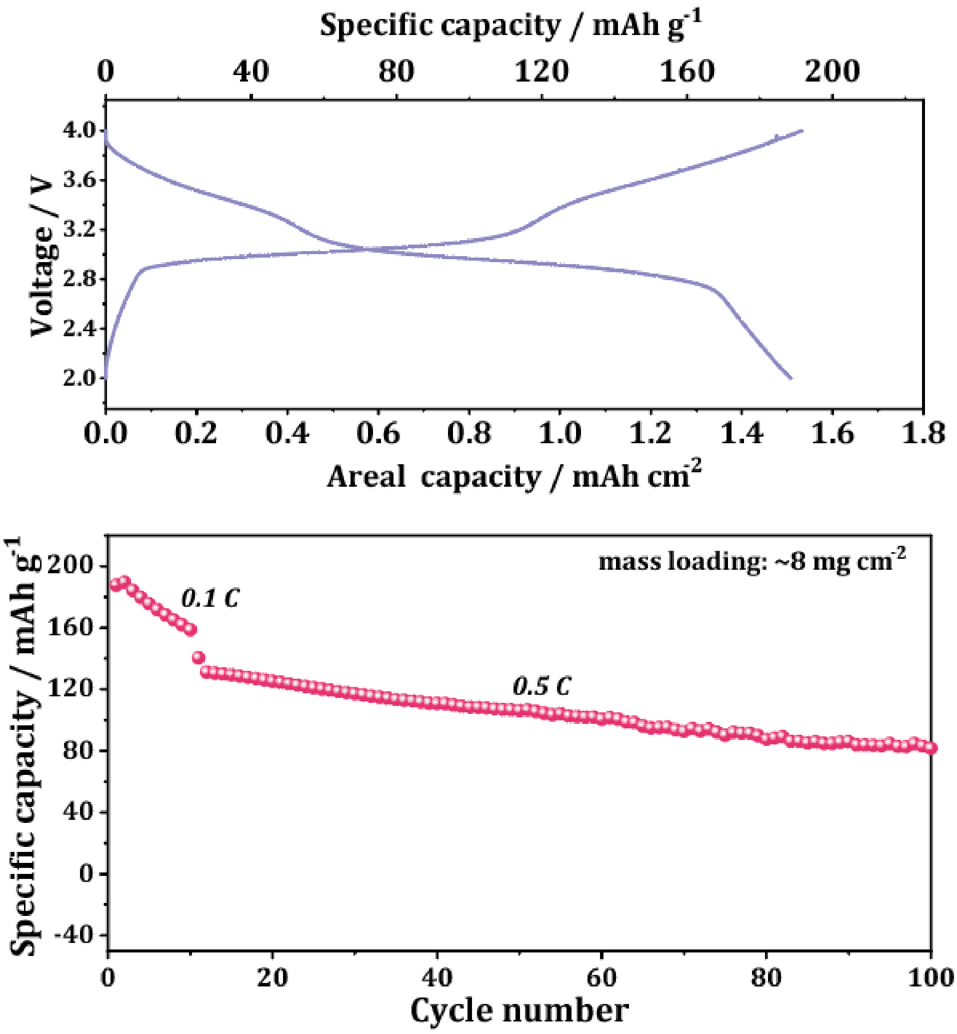

Supplementary Fig. 13 (a) Voltage profile and (b) long-term cycling stability of the all-solid-state LIB at a high I_2 mass loading of ~8 mg cm^{-2}. The battery showed a high areal capacity of 1.5 mAh cm^{-2} at 0.1 C.

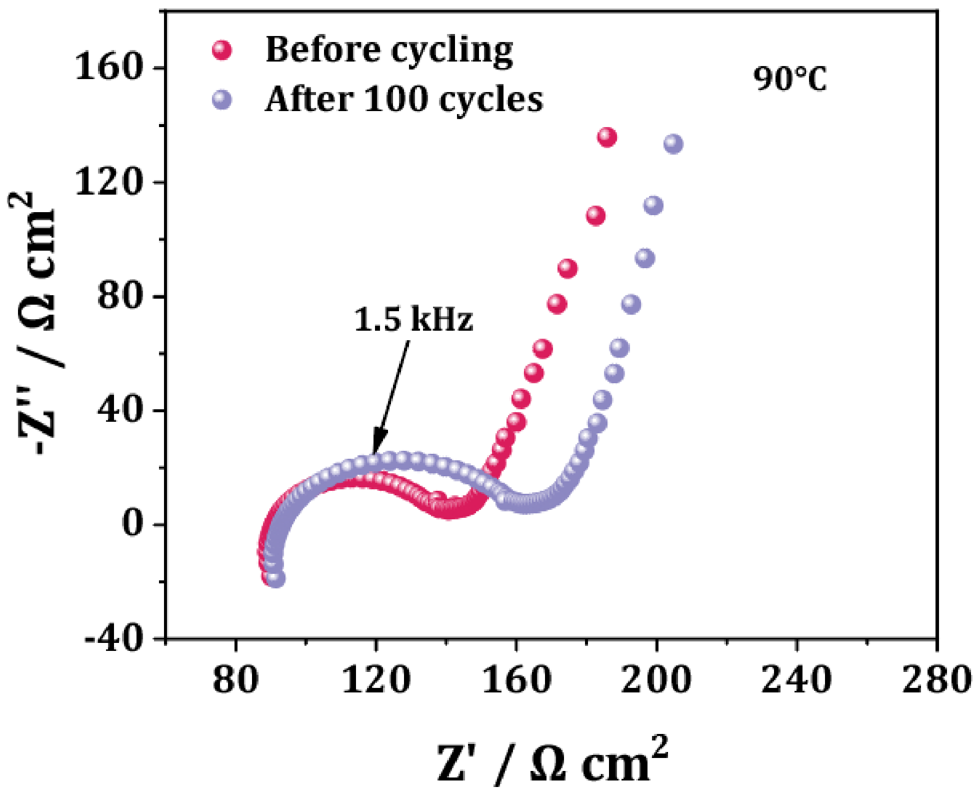

Supplementary Fig. 14 EIS results of the all-solid-state LIB before cycling and after 100 cycles at 90°C.

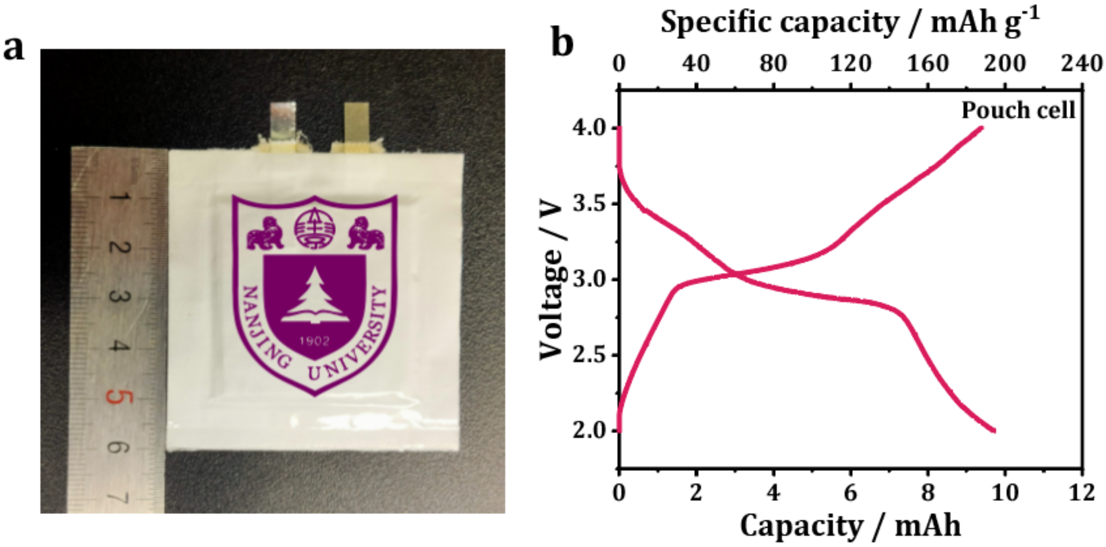

Supplementary Fig. 15 (a) Digital photo and (b) the voltage profile of the as-assembled 40 mm × 40 mm all-solid-state Li-I_2 pouch cell. The cell showed a high capacity of 10 mAh, corresponding to a specific capacity of 190 mAh g^{-1}. This single layer pouch cell is composed of a 4 × 4 cm cathode (210.5 mg total mass, 52.6 mg I_2 mass)", a 4 × 4 cm hybrid electrolyte of 326 μm thick and a 4 × 4 cm Li foil of 100 μm thick.

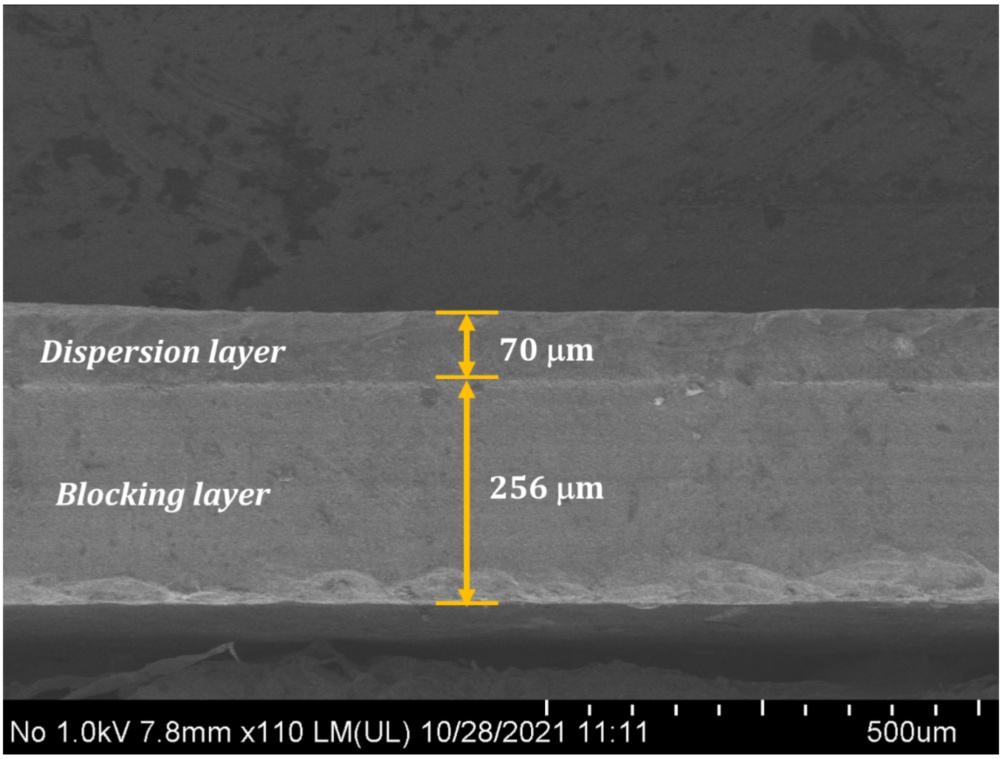

Supplementary Fig. 16 The cross-section SEM image of the hybrid electrolyte. The thickness was measured to be 70 μm for the dispersion layer and 256 μm for the blocking layer.

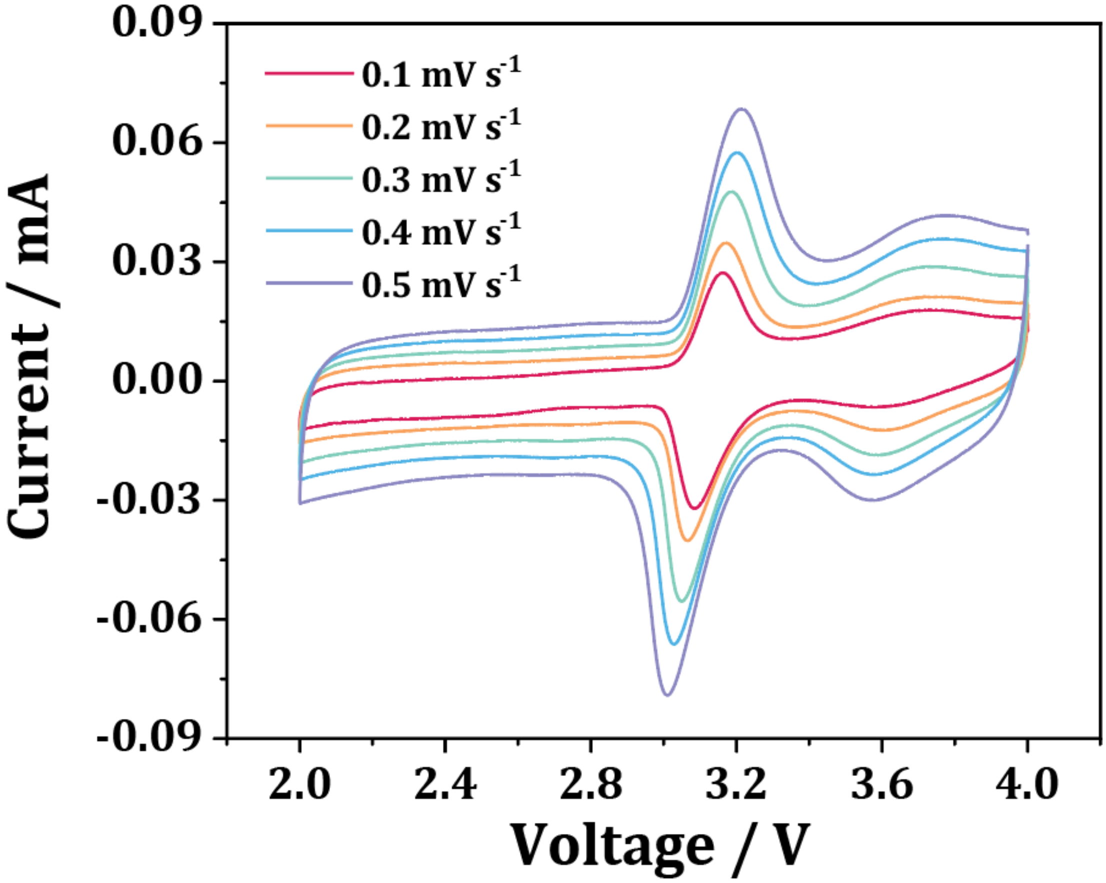

Supplementary Fig. 17 CV curves at sweeping rates of 0.1, 0.2, 0.3, 0.4 and 0.5 mV s^{-1}, showing that the all-solid-state LIB has a highly reversible electrochemical process at various scan rates.

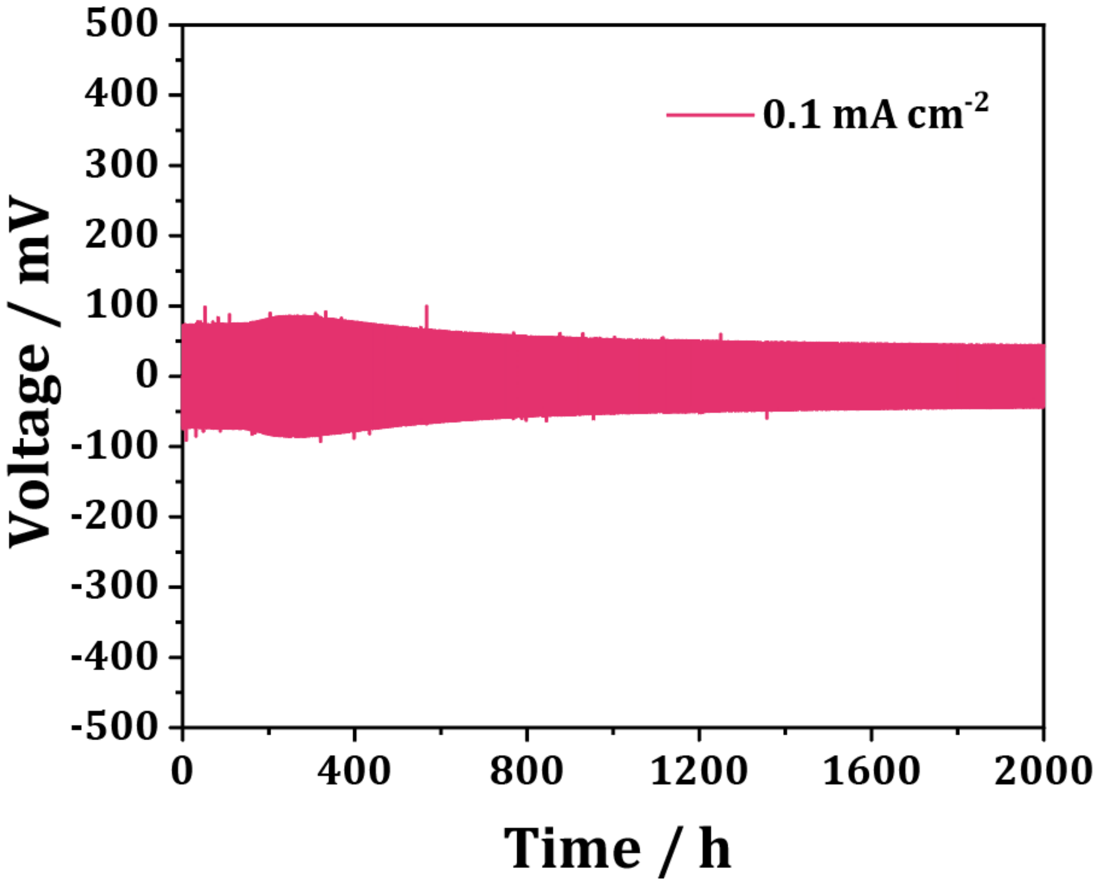

Supplementary Fig. 18 Li-Li symmetric cell based on the hybrid electrolyte showing a low overpotential of 40 mV after 2000 h and an excellent stability at 0.1 mA cm^{-2}. This proves a very stable hybrid electrolyte structure to Li metal anode.

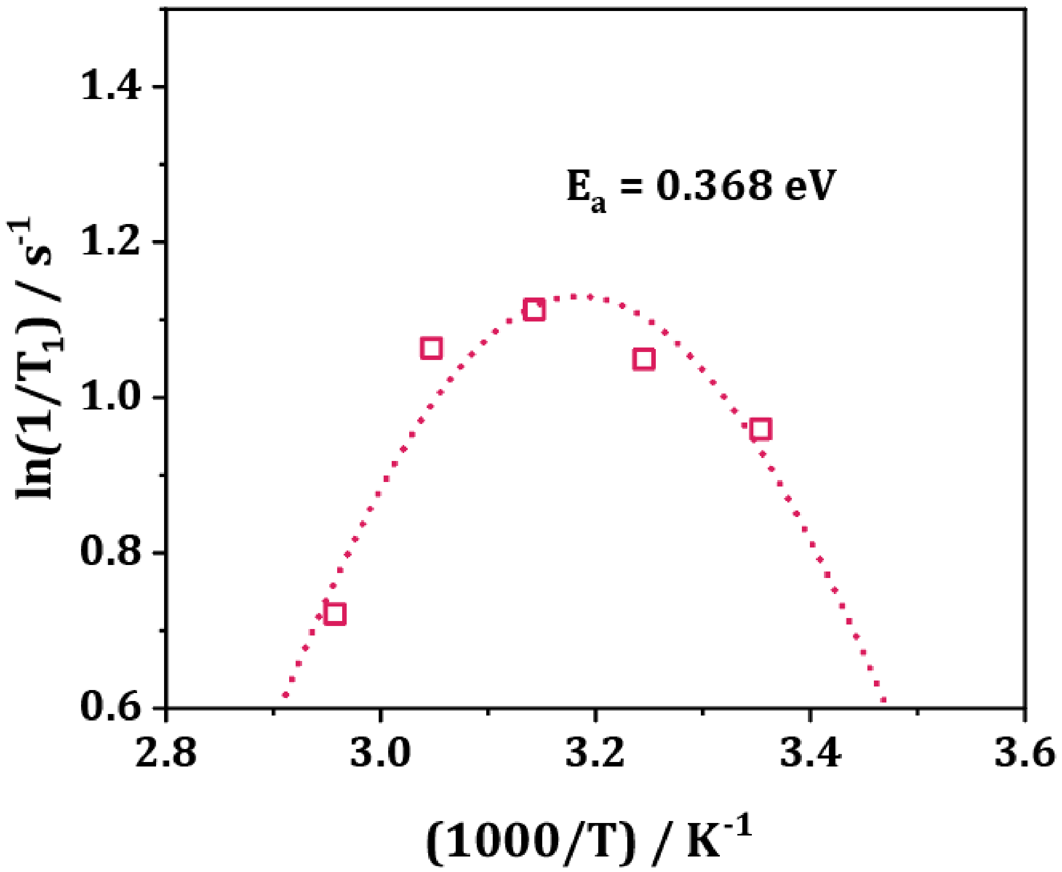

Supplementary Fig. 19 ^{7}Li NMR T_1 relaxation measurement and corresponding fitting of LAGP. The activation energy of Li ion transport in LAGP was calculated to be 0.368 eV, close to the value calculated from EIS result.

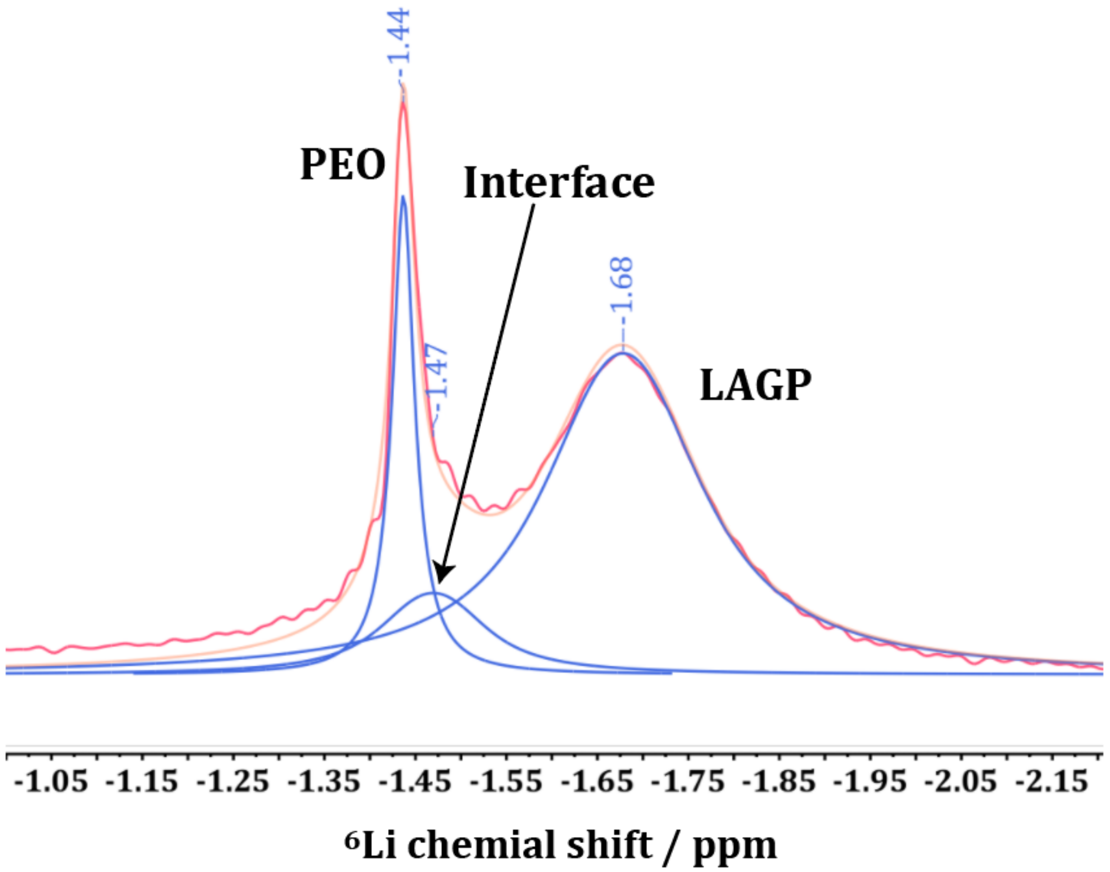

Supplementary Fig. 20 Solid-state ^{6}Li NMR 1D spectrum of hybrid electrolyte sample at a spin rate of 10 kHz. A resonance corresponding to the interface area located at -1.47 ppm appeared between the resonance of PEO (-1.44 ppm) and LAGP (-1.68 ppm).

Supplementary Table 1 | Fitting results of the EIS curves corresponding to different state of discharge/charge shown in Fig. 4d. R_{he} represented the electrolyte bulk resistance, while R_{if} and R_{ct} represented the interfacial resistance and charge transfer resistance respectively.

SOD/SOC	R_{he} / Ω cm^2	R_{if} / Ω cm^2	R_{ct} / Ω cm^2
a	146.6	390.3	671.8
b	146.85	384.9	648.9
c	147.12	383.0	626.5
d	146.85	388.2	607.2
e	145.41	385.4	570.5
f	144.4	383.3	542.3
g	145.47	388.5	543.4
h	144.88	384.8	578.5
i	146.22	383.8	616.2
j	146.57	381.4	622.1
k	147.37	388.2	627.1
l	147.45	382.6	668.1
